# Electroacupuncture remediates glial dysfunction and ameliorates neurodegeneration in the astrocytic α-synuclein mutant mouse model

**DOI:** 10.1186/s12974-015-0302-z

**Published:** 2015-05-28

**Authors:** Jiahui Deng, E Lv, Jian Yang, Xiaoli Gong, Wenzhong Zhang, Xibin Liang, Jiazeng Wang, Jun Jia, Xiaomin Wang

**Affiliations:** Departments of Neurobiology and Physiology, Key Laboratory for Neurodegenerative Disorders of the Ministry of Education, Beijing Key Laboratory for Parkinson’s Disease, Capital Medical University; Beijing Institute for Brain Disorders, Beijing, 100069 China; Department of Neurology and Neurological Sciences, Stanford University, Stanford, CA 94305 USA

**Keywords:** Astrogliosis, α-synuclein, Electroacupuncture, Glial dysfunction, Nrf2

## Abstract

**Background:**

The acupuncture or electroacupuncture (EA) shows the therapeutic effect on various neurodegenerative diseases. This effect was thought to be partially achieved by its ability to alleviate existing neuroinflammation and glial dysfunction. In this study, we systematically investigated the effect of EA on abnormal neurochemical changes and motor symptoms in a mouse neurodegenerative disease model.

**Methods:**

The transgenic mouse which expresses a mutant α-synuclein (α-syn) protein, A53T α-syn, in brain astrocytic cells was used. These mice exhibit extensive neuroinflammatory and motor phenotypes of neurodegenerative disorders. In this study, the effects of EA on these phenotypic changes were examined in these mice.

**Results:**

EA improved the movement detected in multiple motor tests in A53T mutant mice. At the cellular level, EA significantly reduced the activation of microglia and prevented the loss of dopaminergic neurons in the midbrain and motor neurons in the spinal cord. At the molecular level, EA suppressed the abnormal elevation of proinflammatory factors (tumor necrosis factor-α and interleukin-1β) in the striatum and midbrain of A53T mice. In contrast, EA increased striatal and midbrain expression of a transcription factor, nuclear factor E2-related factor 2, and its downstream antioxidants (heme oxygenase-1 and glutamate-cysteine ligase modifier subunits).

**Conclusions:**

These results suggest that EA possesses the ability to ameliorate mutant α-syn-induced motor abnormalities. This ability may be due to that EA enhances both anti-inflammatory and antioxidant activities and suppresses aberrant glial activation in the diseased sites of brains.

## Background

As a conventional cytosolic protein, α-synuclein (α-syn), has been assumed to exert its pathogenic function exclusively in the cytoplasm in a cell-autonomous manner [1]. However, this view has been challenged by the presence of α-syn and its aggregated forms in the extracellular fluid such as cerebrospinal fluid and blood plasma of both Parkinson’s disease (PD) and normal subjects [2, 3]. Although the mechanisms underlying α-syn secretion are not fully understood, evidence has shown that α-syn fibrils are released to the extracellular environment by non-classical exocytosis [4, 5]. Extracellularly misfolded α-syn can propagate in a prion-like spreading fashion [6, 7] and be endocytosed by neighboring neurons or glia [8], through which α-syn spreads and amplifies degenerative signals from cells to cells [9].

Glial cells are particularly involved in cell-to-cell contacts of α-synucleinopathies in the progression of neurodegenerative disorders [10]. Several lines of evidence have shown that α-syn-containing inclusion bodies aggregated in astrocytes and oligodendrocytes in PD, dementia with lewy bodies, and multiple system atrophy [11–13]. In fact, α-syn-positive inclusions have been observed in astrocytes and oligodendrocytes in the substantia nigra (SN) and/or locus coeruleus of postmortem PD patients, and the number of glial α-syn-inclusions was related to the progress of PD [14]. However, the underlying mechanisms for α-synucleinopathies in glia and its roles in disease onset and progression remain unclear.

An experimental α-syn-inducible transgenic mouse, in which PD-related A53T mutant α-syn is selectively expressed in astrocytes, developed an early-onset, rapidly progressed movement disability. Increasing astrocytic α-syn deposition initiated widespread astrogliosis, microglial activation, and dopaminergic neuronal degeneration [15]. It appears that α-syn induces early astrocytic dysfunction and severe inflammation responses, which accelerate neuronal damage and PD progression [10]. In addition, α-syn is released from neuronal cells, and the released α-syn is readily endocytosed by astrocytes, leading to neuroinflammatory responses [16]. Therefore, restoring glial function and inhibiting microglial activation are of therapeutic benefit for treating PD [17].

Due to severe side effects induced by long-term medications for treating chronic neurodegenerative diseases, it is necessary to find non-pharmacological tools to ameliorate these disorders. Acupuncture or electroacupuncture (EA) represents a non-invasive procedure and has long been used to relieve pain and promote stroke rehabilitation [18, 19]. Several neurodegenerative disorders, such as amyotrophic lateral sclerosis [20], ischemia [21], and PD [22, 23], are also seemingly suitable for EA treatment. Although the use of EA has been endorsed by the US National Institutes of Health and the World Health Organization, and the anti-inflammatory effect of acupuncture has been well documented in human and animal studies [24–27], little is understood about its biochemical basis for therapy.

In this study, we attempted to investigate the neurochemical basis for the therapeutic effect of EA. To this end, we used a transgenic mouse line which was constructed to selectively express α-syn A53T mutant in astrocytic cells. The anti-inflammatory effect of EA was investigated in these mice. The effects of EA on motor behavior and activities of glial and neuronal cells were also examined. In addition, the nuclear factor E2-related factor 2 (Nrf2)-antioxidant response element (ARE) signaling pathway, a key mediator in oxidative stress and inflammation [28], was analyzed for its responses to EA.

## Methods

### Animals

TetO-A53T mice, tetracycline operator (tetO)-regulated α-syn transgenic mice, and glial fibrillary acidic protein (GFAP)-tTA mice (GFAP promoter-controlled tetracycline transactivator transgenic mice) were obtained from Prof. Huaibin Cai’s laboratory in the National Institutes of Health. These mice were crossed to generate GFAP-tTA/tetO-α-syn (A53T) double transgenic mice. The mice were housed in a pathogen-free climate-controlled facility with diet and water available *ad libitum*. The animal room was on a 12/12 h light/dark cycle. To assess the impact of developmental expression of A53T α-syn on the neurological function, a cohort of A53T mice was administered with doxycycline (DOX), a tetracycline derivative, to block the expression of A53T α-syn from the embryonic stage to postnatal day 21 (P21). Genotypes were determined by polymerase chain reaction (PCR) analysis of tail DNA [15]. All procedures were approved by the Ethical Committee for Animal Research of Capital Medical University.

### EA stimulation

Mice were divided into four groups: untreated non-transgenic (nTg) control, untreated A53T transgenic, 100 Hz EA-treated nTg, and 100 Hz EA-treated A53T mice. The EA stimulation was given for 4 weeks starting at 2 months of age. Mice were gently handled and lightly restrained in a plastic cylinder (7 × 2.5 cm) with their hind limbs accessible for needling. For this procedure, the mice were immobilized for 30 min during EA administration with no anesthetization [29]. After cleaning the skin with alcohol, two sterilized stainless-steel needles (0.18 mm diameter × 3 mm length) were inserted in each leg, one at ST36 (2 mm lateral to the anterior tubercle of tibia) and the other at SP6 (2 mm proximal to the upper border of medial malleolus, at the posterior border of the tibia). The EA performed at these acupoints is known to relieve PD-like symptoms such as muscle and movement disorders [30]. The stimuli were generated from a Han’s Acupoint Nerve Stimulator (HANS, LH series, manufactured in Peking University). Bidirectional square wave electrical pulses (0.2 ms duration, 100 Hz) designated as EA were given for a total of 30 min each day. The intensity of the stimulation at 100 Hz was increased stepwise from 1 to 1.2 mA and then to 1.4 mA, with each step lasting for 10 min.

### Behavioral test

#### Open-field test

Locomotor activity was assessed in automated activity chambers (25.4 × 25.4 in. square) connected to a digital scan analyzer that transmitted the number of infrared beam breaks (activity data) to a Mouse Tru Scan system (Truscan 2.0 Instruments, Columbus, USA). Total movement distance (cm) was recorded across a 30-min recording period.

#### Rotarod test

The length of time a mouse stayed on a rotating rod with auto acceleration from 0 rotation per minute (rpm) to 40 rpm in 5 min (Acceler ROTA-ROD, Jones & Roberts, CA) was recorded.

#### Grip strength measurement

Mouse forepaws or hindpaws were pulled or compressed a triangular bar linked to a digital force gauge (San Diego Instruments, San Diego, CA). The maximal pulling or compressing force was recorded. The mean of five measurements was analyzed for each animal during each test.

#### Gait analysis

The Catwalk gait analysis method has been reported in details [31]. Briefly, mice were placed individually in the Catwalk walkway system (Noldus Information Technology, The Netherlands) and were allowed to walk freely and traverse from one side to the other of the walkway glass plate. During the recording, the environment was kept completely dark, except for the light from the computer screen. Where the mouse paws made contact with the glass plate, light emitting diode light was reflected down and the illuminated contact areas were recorded with a high speed color video camera underneath the glass plate connected to a computer that run the Catwalk software 9.1. Stride length and interlimb coordination measured as regularity index, which is the percentage of normal step sequences, were analyzed.

### Histology and immunohistochemical analysis

Mice were deeply anesthetized and transcardially perfused with saline followed by fixative (4 % paraformaldehyde in 0.1 M phosphate buffered saline (PBS), pH 7.4. Brain and spinal cord tissues were removed and post-fixed in the same fixative overnight. Following equilibration in 30 % sucrose in PBS, 30 μm sections were cut in the coronal plane with a cryostat at −20 °C and processed for immunohistochemistry. Primary antibodies against GFAP (1:1000, Sigma-Aldrich USA, St. Louis, MO), ionized calcium-binding adaptor molecule-1 (Iba1) (1:1000, Wako Chemicals USA, Richmond, VA), tyrosine hydroxylase (TH) (1:1000, Sigma-Aldrich USA, St. Louis, MO), α-syn (C20 & 211, 1:1000, Santa Cruz Biotech, Santa Cruz, CA), and secondary Alexa 488- or Alex 568-conjugated antibodies (1:1000, Invitrogen) were used to visualize the staining. Dapi (1:1000, Invitrogen) was used for counterstaining the nuclei. A monoclonal mouse anti-neuronal Nuclei (NeuN) antibody was used at a 1:1000 dilution (Chemicon International, Temecula, CA). Immunofluorescent images were captured using a laser scanning confocal microscope (Leica TCS SP8, Germany).

### Stereology

According to stereotaxic coordinates of mouse brains [32], a series of coronal sections across the substantia nigra pars compacta (SNpc) (Bregma −2.54 ~ −3.88 mm) was stained with TH and visualized using the Vectastain ABC kit (Vector Laboratories, Burlingame, CA). The number of TH-positive cells was assessed using an unbiased stereological procedure with an optical fractionator Stereo Investigator (Micro-Bright Field Inc, Williston, VT). The coefficient of error (CE) was designed less than 10 % in order to get reliable results.

### Quantitation of proinflammatory cytokines

We quantified proinflammatory cytokine levels in the SNpc and striatum. Tissues were homogenized in ice-cold lysis buffer containing 137 mM NaCl, 20 mM Tris (pH 8.0), 1 % (*v/v*) glycerol, 1 % (*v/v*) Nonidet P-40, 1 mM phenylmethylsulfonyl fluoride, and 0.5 mM sodium vanadate. The homogenate was centrifuged at 1500*g* for 15 min at 4 °C. The supernatant was collected and stored at −80 °C before use. The levels of tumor necrosis factor-α (TNF-α) and interleukin-1β (IL-1β) were detected using mouse TNF-α and IL-1β enzyme-linked immunosorbent assay (ELISA) kits (Shanghai ExCell Biology Inc., Shanghai, China), respectively, according to the manufacturer’s instructions. The sensitivity of the ELISA assay was 15 pg/ml for both TNF-α and IL-1β. Standards were assayed in duplicates. The protein concentration was determined using a detergent compatible protein assay with bovine serum albumin as the standard.

### Western blot

Proteins were separated on 10 % SDS-PAGE gels and electrophoretically transferred to polyvinylidene difluoride membranes (Millipore). Membranes were blocked with 5 % nonfat dry milk in PBS for 2 h at room temperature and incubated overnight at 4 °C with a primary antibody. Antibodies against α-syn (1:1000, Santa Cruz Biotech, Santa Cruz, CA), Nrf2 (1:500, Sigma-Aldrich USA, St. Louis, MO), heme oxygenase-1 (HO-1) (1:300, Abcam, Cambridge, UK), glutamate-cysteine ligase modifier subunits (GCLM) (1:500, Abcam, Cambridge, UK), or β-actin (1:5000, Sigma-Aldrich USA, St. Louis, MO) were used in this study. The membranes were then treated with an anti-rabbit or anti-mouse antibody conjugated with IRDye™ 800 green or IRDye™ 700 red (Rockland, Limerick, PA) for 1 h. They were washed three times with PBS containing 0.1 % Tween and twice with PBS alone. Proteins were visualized by a LI-COR Odyssey infrared double-fluorescence imaging system (American Company LICOR).

### High-performance liquid chromatography (HPLC)

Samples from striatal tissue were prepared to detect the DA concentration as previously described [29]. Each sample was injected into an HPLC system (Model 5600A; CoulArray Detector System ESA, Brighton, MA) for analysis. The mobile phase was 0.125 M sodium citrate buffer containing 20 % methanol, 0.1 mM Na_2_EDTA, and 0.5 mM 1-octanesulfonic acid (Acros Organics, Morris Plains, NJ).

### Isolation of total RNA and quantitative real-time PCR

The isolation of total RNA was performed by using an RNA NucleoSpin® kit according to the manufacturer’s instructions (Macherey-Nagel, Germany). Reverse transcriptase reactions were run on 1 μg of total messenger RNA (mRNA) by using ProtoScript M-Mulv First Strand cDNA Synthesis Kit (New England Biolabs, USA). Quantitative PCR was performed by using a Brilliant IISYBR Green QPCR Master Mix (Agilent Technologies, USA). The primers sequences were α-synF (5′-TGG ATG TAT TCA TGA AAG GA-3′), α-synR (5′-CCA GTG GCT GCT GCA ATG CTC-3′), GFAPF (5′-CGA GTC CCT AGA GCG GCA AAT G-3′), GFAPR (5′-CGG ATC TGG AGG TTG GAG AAA GTC-3′), Nrf2/5′ (5′-TTC TTT CAG CAG CAT CCT CTC CAC-3′), Nrf2/3′ (5′-ACA GCC TTC AAT AGT CCC GTC CAG-3′), HO-1/5′ (5′-CAA GCC GAG AAT GCT GAG TTC ATG-3′), HO-1/3′ (5′-GCA AGG GAT GAT TTC CTG CCA G-3′), GCLM/5′ (5′-GCC ACC AGA TTT GAC TGC CTT TG-3′), GCLM/3′ (5′-TGC TCT TCA CGA TGA CCG AGT ACC-3′), ActinF (5′-CTG GCT CCT AGC ACC ATG AAG ATC-3′), ActinR (5′-TGC TGA TCC ACA TCT GCT GG-3′).

### Statistical analysis

All data are represented as means ± SEM and were analyzed by one-way ANOVA or Student’s *t* test as appropriate by using Prism 5.0 software (GraphPad Software, San Diego, CA). *P* < 0.05 was considered statistically significant.

## Results

### EA delayed the early onset of movement disability in A53T mice

**Fig. 1:**
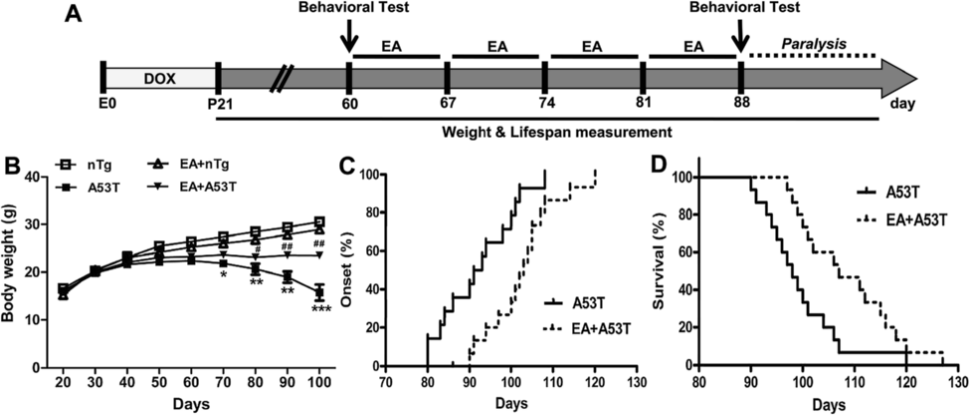
EA reduced loss of body weight, delayed paralysis onset, and increased lifespan in A53T mice. **a** Schematic diagram illustrating the experimental timeline for behavioral tests and EA treatments. **b** Effects of EA on the loss of body weight in A53T mice (*n* = 10 per group). Note a significant loss of body weight starting at 70 days in A53T mice. EA was able to reverse the loss at three testing days (80, 90, and 100 days). **c** Effects of EA on the onset of paralysis observed in A53T mice (*n* = 15 per group). **d** Effects of EA on survival rate of A53T mice (*n* = 15 per group). Data in Fig. 1b are expressed as means ± SEM. *P < 0.05, **P < 0.01, ***P < 0.001 vs. nTg group. **#** *P* < 0.05, #**#** *P* < 0.01 vs. A53T group

A53T α-syn mice were administered with DOX from embryonic stages (E0) to P21 to block the developmental expression of A53T α-syn (Fig. 1a). At day 60 (P60), A53T or nTg control mice received 4-week EA treatment. Before and after EA treatment, motor behaviors were examined.

A53T mice displayed a loss of body weight as compared to age-matched nTg mice at 70th day. The body weight of A53T mice continued to drop during the disease progression. EA at 100 Hz significantly prevented the loss of body weight. However, EA had no effect on the body weight of nTg mice (Fig. 1b). The early-onset paralysis was also seen in A53T mice which may result from the significant neuronal loss in the midbrain and spinal cord [15]. Average age of onset for paralysis developed with one of four limbs in A53T mice was 92.9 ± 2.3 days. This symptom then quickly spread to other limbs which eventually caused death of animals at an average lifespan of 99.4 ± 2.0 days (Fig. 1c, d). Interestingly, 100 Hz EA significantly delayed the average age of onset to 102.7 ± 2.1 days and prolonged the average lifespan to 108.9 ± 2.3 days (Fig. 1c, d).

**Fig. 2:**
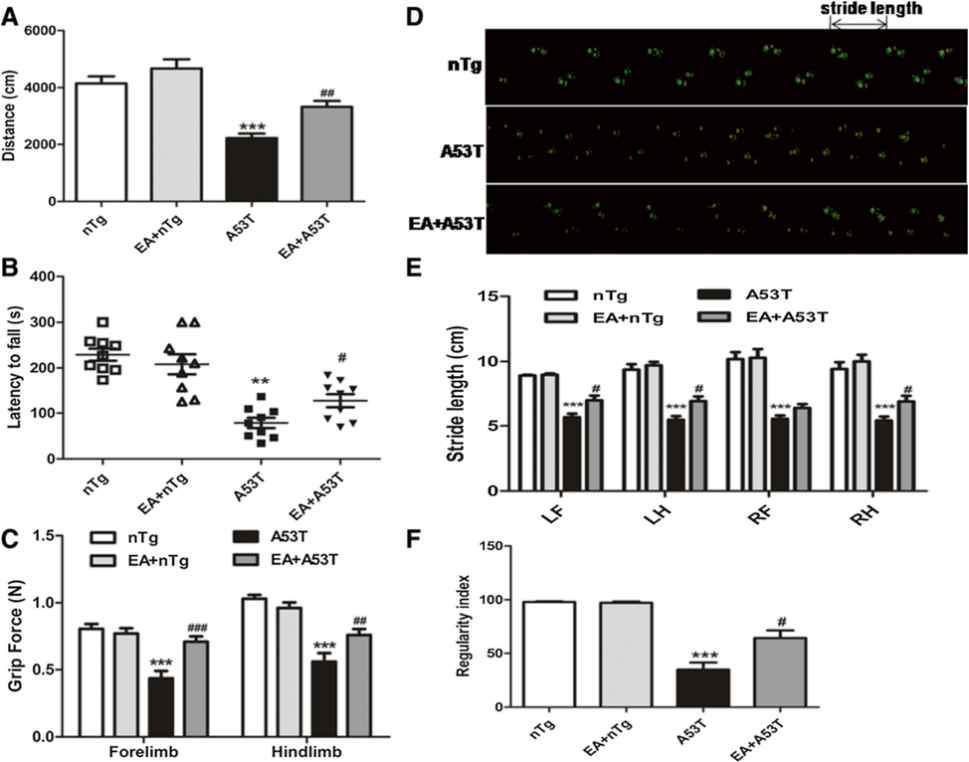
EA improved motor function in A53T mice. **a** Effects of EA on total distance in A53T and control mice. Mice in each group were recorded for 30 min (*n* = 9 per group). **b** Effects of EA on the duration of A53T and control mice stayed on the rotating rod (*n* = 9 per group). **c** Effects of EA on the grip strength of forelimbs and hindlimbs in A53T and control mice (*n* = 9 per group). **d** and **e** Effects of EA on the stride length of four limbs in A53T and control mice (*n* = 6 per group). **f** Effects of EA on the regularity index of A53T and control (*n* = 6 per group). Note that EA stimulation generally improved all motor activities surveyed. Data are expressed as means ± SEM. ***P < 0.001 vs. nTg group. #*P* < 0.05, ##*P* < 0.01, ###*P* < 0.001 vs. A53T group

To examine the effect of EA on motor behaviors, open-field test was first carried out to assess the spontaneous locomotor activity in A53T and nTg mice after 4-week EA treatment. The movement distance was significantly decreased in A53T mice (2221.7 ± 160.0 cm) compared to nTg mice (4145.9 ± 252.3 cm). EA at 100 Hz effectively increased the movement distance (3320.1 ± 211.0 cm) (Fig. 2a). The motor coordination of A53T mice was decreased in a rotarod test, which was partially reversed by 100 Hz EA (Fig. 2b). The grip strength test is a feasible way to objectively quantify the muscular strength of rodents. The mean grip force in the fore- and hindlimbs of A53T mice was significantly reduced relative to nTg mice (Fig. 2c). EA (4 weeks, 100 Hz) restored the mean grip force by 62.9 and 35.6 % respectively in the fore- and hindlimbs compared to A53T mice without EA (Fig. 2c). In addition, Catwalk gait analysis showed a significant decrease in stride length of all four paws in A53T mice. One hundred hertz EA significantly increased its stride length in the left-fore, left-hind, and right-hind paw although not the right forelimb (Fig. 2d, e). The interlimb coordination measured by regularity index in A53T mice was reduced to 47.7 % of control nTg mice. One hundred hertz EA recovered the regularity index by 84.6 % during the course of the experiment (Fig. 2f). EA had no significant effect on all motor activities surveyed in nTg mice.

Noticeably, within 2 months of age, EA had a minimal impact on the motor impairments in movement distance, latency time, grip force, and Catwalk gait (data not shown). After 2 months of age, A53T mice showed an acceleration of the motor disability. At the same time, EA treatment had its maximal effects. The acceleration of the motor disability after 2 months might be due to substantial expression of α-syn in astrocytes. EA by targeting overexpressed α-syn could therefore exert its therapeutic effects (see below).

### EA alleviated astrocytic α-syn expression in A53T mice

**Fig. 3:**
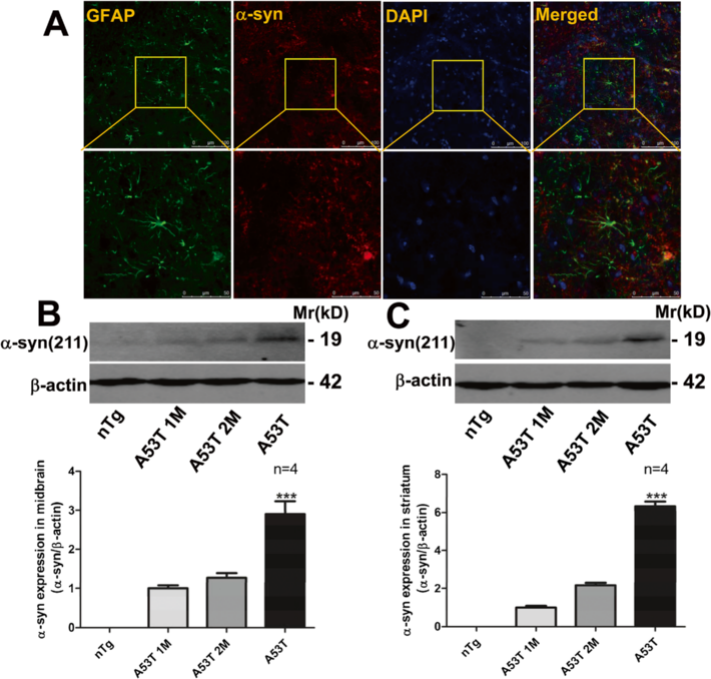
Expression of α-syn in astrocytes in the SN and striatum of A53T mice. **a** Representative immunofluorescent photomicrographs showing α-syn (*red*) and GFAP (*green*) costaining in the SN of A53T mice. The α-syn immunoreactivity was restricted to GFAP-expressing astrocytes. **b** and **c** Immunoblot analysis of expression of α-syn proteins in the midbrain and striatum of A53T mice. Representative immunoblots are shown above the quantified data. A53T mice were sacrificed for subsequent immunoblot analysis at 1 or 2 months after the birth or at the paralysis development in the symptomatic A53T mice. Data are expressed as means ± SEM (*n* = 4 per group). ***P < 0.001 vs. 1 or 2 months

**Fig. 4:**
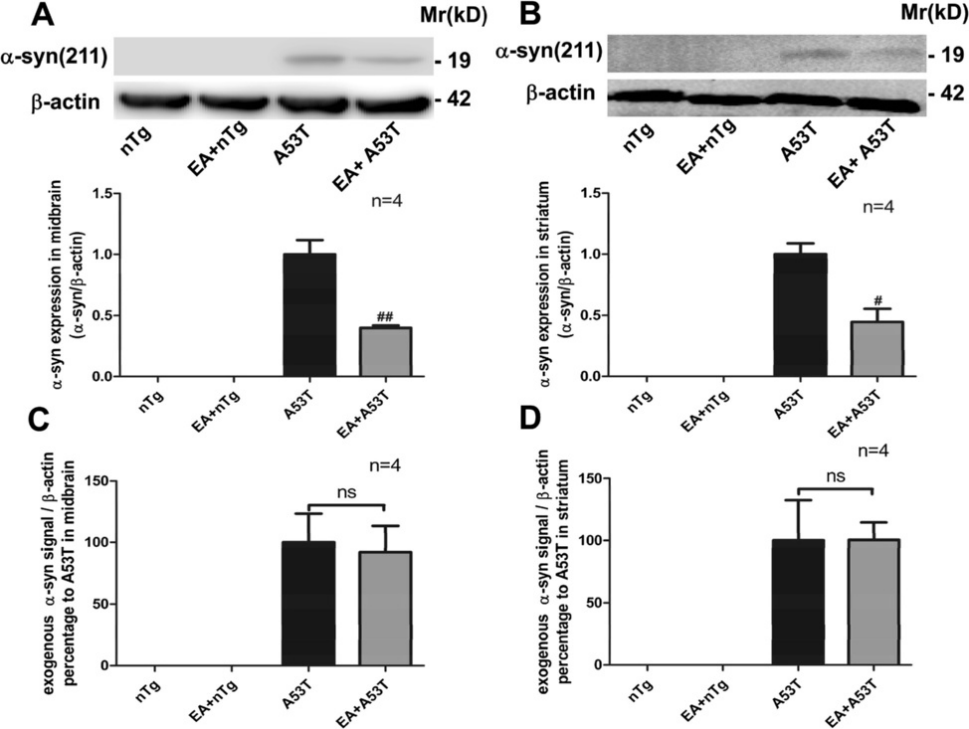
EA decreased exogenous α-syn expression in the midbrain and striatum of A53T mice. **a** and **b** Effects of EA on α-syn protein expression in the midbrain (**a**) and striatum (**b**) of A53T and nTg control mice. Representative immunoblots are shown above the quantified data. Note that expression level of α-syn proteins in both the midbrain and striatum was markedly reduced by EA. **c** and **d** Effects of EA on α-syn mRNA expression in the midbrain (**c**) and striatum (**d**) of A53T and nTg control mice. The α-syn mRNA level in midbrain and striatal tissue was detected by real-time PCR. Data are expressed as means ± SEM (*n* = 4 per group). #*P* < 0.05, ##*P* < 0.01 vs. A53T group

To observe the influence of EA on the aggregation of α-syn, expression of A53T α-syn in the midbrain and striatum was investigated in this study. Abundant expression of A53T α-syn was seen in the GFAP-positive astrocytes as demonstrated by co-staining of α-syn with GFAP in the SN (Fig. 3a). Western blots also showed that the level of exogenous α-syn was gradually increased in the midbrain and striatum over different time points (1 month, 2 month, and symptomatic time) in A53T mice, while α-syn immunoreactivity was absent in these regions in nTg control mice (Fig. 3b, c). After 4 weeks of 100 Hz EA treatment, the level of α-syn proteins in the midbrain of A53T mice became significantly lower than that in untreated A53T mice (Fig. 4a). Similar results were observed in the striatum (Fig. 4b). In contrast to proteins, EA did not affect α-syn mRNA expression in the midbrain (Fig. 4c) and striatum (Fig. 4d). In nTg mice with or without EA, no α-syn expression at either protein or mRNA levels was observed. These data suggest that 100 Hz EA, while it did not affect A53T α-syn transcription, downregulated α-syn protein expression in the midbrain, and striatum of mutant mice.

### EA attenuated astrogliosis in A53T mice

**Fig. 5:**
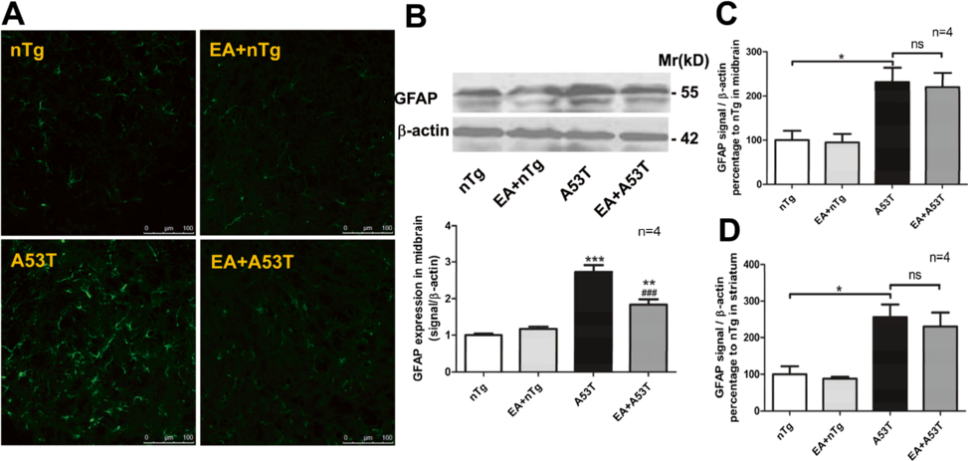
EA reduced the astrogliosis in the midbrain of A53T mice. **a** Immunofluorescent images illustrating effects of EA on GFAP immunostaining in the SN of A53T and control nTg mice. GFAP-containing astrocytes were visualized by GFAP immunostaining. **b** Immunoblots illustrating the effects of EA on GFAP protein expression in the midbrain. Representative immunoblots are shown above the quantified data. **c** and **d** Effects of EA on GFAP mRNA expression in the midbrain (**c**) and striatum (**d**) of A53T and control nTg mice. α-syn mRNAs were detected determined by quantitative real-time PCR. Data are expressed by means ± SEM (*n* = 4 per group). **P < 0.01, ***P < 0.001 vs. nTg group. #*P* < 0.05, ###*P* < 0.001 vs. A53T group

Astrocytic expression of A53T α-syn appeared to disrupt multiple normal functions of astrocytes, resulting in severe astrogliosis throughout the brain and spinal cord [15]. We then assessed the rate of astrogliosis by analyzing expression of GFAP in A53T mice. Immunofluorescent staining revealed a larger number of GFAP-positive astrocytes in the SN of A53T mice (Fig. 5a). EA (100 Hz) decreased the number of GFAP-immunoreactive astrocytes (Fig. 5a). Similarly, in immunoblot assays, EA effectively alleviated the excessive expression of GFAP in the SN of A53T mice (Fig. 5b). Of note, the level of GFAP in the EA-treated A53T mice still remained higher than that seen in control nTg mice. In addition, GFAP mRNA expression was probed using quantitative RT-PCR. We found that GFAP mRNA expression was elevated in the midbrain (Fig. 5c) and striatum (Fig. 5d) of A53T mice. However, unlike GFAP proteins, GFAP mRNA levels in these regions remained at a higher control level after EA treatment. The fact that EA limited the protein although no mRNA expression of GFAP in astrocytes of A53T mice indicates that EA may primarily inhibit reactive astrogliosis.

### EA alleviated microglial activation and inflammation responses in A53T mice

**Fig. 6:**
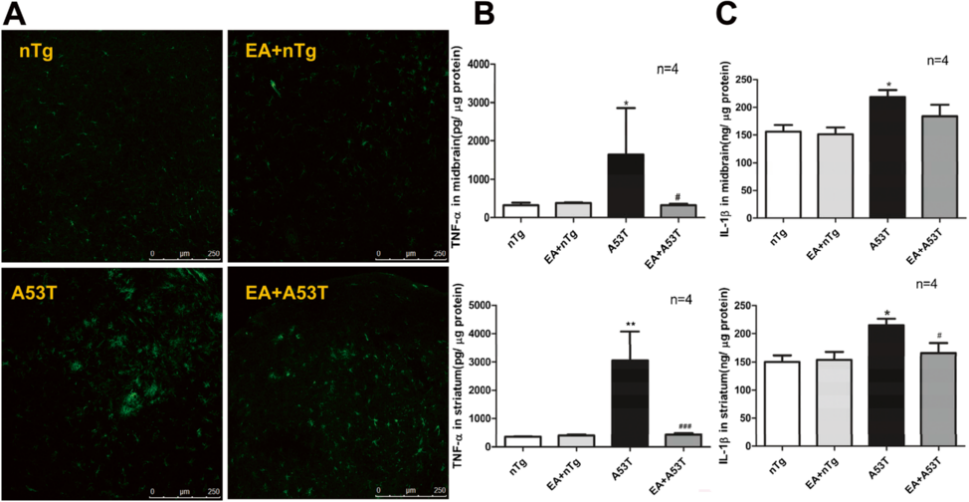
EA inhibited microglia activation and inflammatory responses in A53T mice. **a** Immunofluorescent images illustrating effects of EA on Iba1 immunostaining in the SN of A53T and nTg control mice. Iba1-containing microglia was visualized by Iba-1 immunostaining. **b** and **c** Effects of EA on TNF-α (**b**) and IL-1β (**c**) expression in the midbrain (upper panels) and striatum (lower panels) of A53T mice and nTg control mice. TNF-α and IL-1β protein levels were assessed by ELISA. Note that EA significantly reduced TNF-α expression in the midbrain and striatum and IL-1β expression in the striatum of A53T mice. Data are expressed as means ± SEM. *P < 0.05, **P < 0.01 vs. nTg group. #*P* < 0.05, ###*P* < 0.001 vs. A53T group

Whether EA affects microglial activation and inflammatory responses was investigated in A53T mice. Activated microglia was observed in the SN of A53T mice, as evidenced by increased Iba-1 immunoreactivity and by the appearance of microglia clusters (Fig. 6a). EA stimulation effectively reduced the intensity of Iba-1 immunostaining and the clusters of activated microglia. Levels of inflammatory cytokines, such as TNF-α and IL-1β closely correlating with the presence of activated microglia, were quantified by ELISA in the SN and striatum. A53T mice exhibited a higher level of TNF-α expression in the midbrain and striatum, which was blocked by EA treatment (Fig. 6b). Similar results were seen for IL-1β expression in the striatum (Fig. 6c). Regarding IL-1β expression in the midbrain, an insignificant increase in IL-1β levels was observed in A53T mice after EA treatment. These results demonstrate the ability of EA to inhibit microglial activation and neuroinflammation induced by exogenous expression of A53T α-syn in astrocytes.

### EA prevented dopaminergic neurodegeneration and loss of spinal motor neurons in A53T mice

**Fig. 7:**
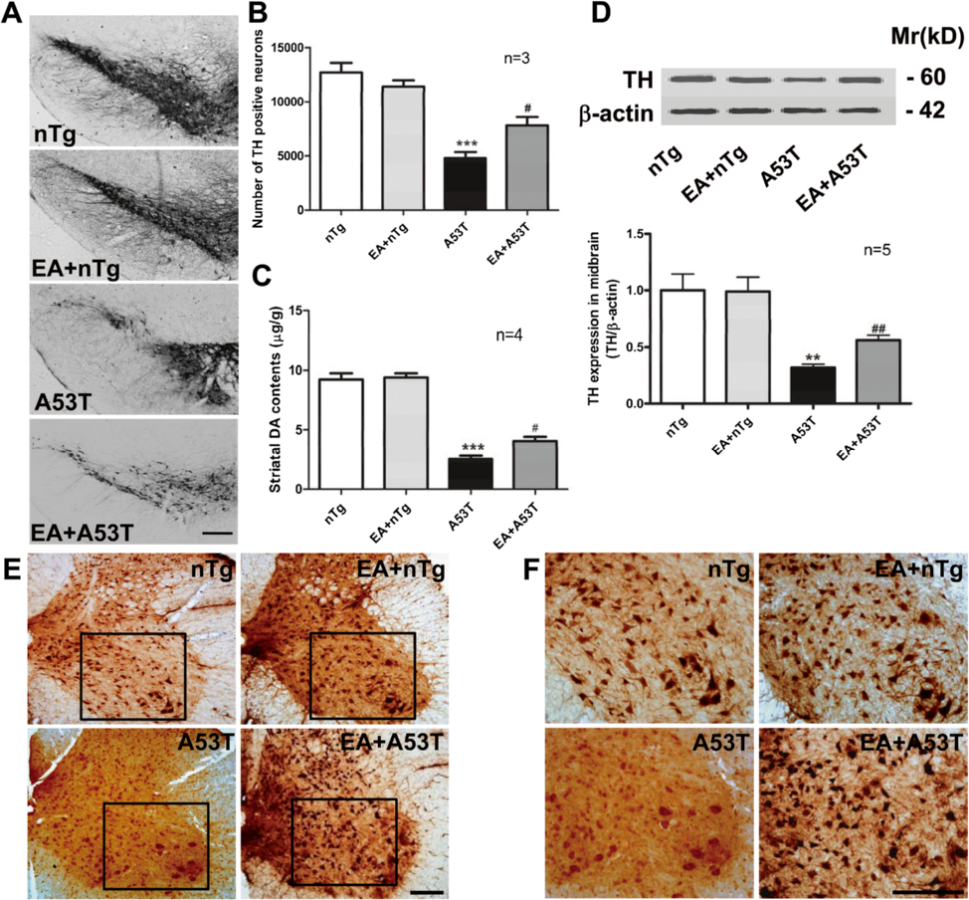
EA protected DA neurons in the SNpc and motor neurons in the spinal cord in A53T mice. **a** Representative immunohistochemical photomicrographs showing TH immunostainings in the SNpc of A53T and nTg control mice following EA treatment. Scale bar, 200 μm. **b** Quantification of TH-positive dopaminergic neurons in the SNpc of A53T and nTg control mice following EA treatment. The TH neurons were counted by stereological analysis. **c** Effects of EA on the striatal DA content. Striatal DA levels were assessed by HPLC. **d** Effects of EA on TH protein levels in the midbrain. TH proteins were detected by immunoblot analysis. Representative immunoblots are shown above the quantified data. **e** Representative images show NeuN staining in the ventral horn of lumbar spinal cord of control nTg mice, EA+ nTg mice, A53T mice, and EA+ A53T mice. Scale bar, 200 μm. **f** These images are the enlarged views from areas indicated in the (**e**). Scale bar, 200 μm. Data are expressed by means ± SEM (*n* = 5 per group). *P < 0.05, **P < 0.01, ***P < 0.001 vs. nTg group. #*P* < 0.05, ##*P* < 0.01 vs. A53T group

To determine whether EA prevents neuronal loss in the midbrain of A53T mice, we monitored changes in the number of TH-positive dopaminergic neurons in the SNpc using an unbiased stereological method. As expected, the number of TH-positive neurons was decreased by 62.4 % in the SNpc of A53T mice compared to nTg mice (Fig. 7a, b). EA treatment significantly rescued the loss of neurons (Fig. 7a, b). A significant reduction of DA content (72.5 % of control) was seen in the striatum of A53T mice as detected by HPLC assays (Fig. 7c). EA stimulation partially reversed this reduction. In addition, reduced TH protein expression in the midbrain of mutant mice was partially recovered by EA (Fig. 7d). Thus, EA promotes survival of dopaminergic neurons in the midbrain of A53T mice.

In addition, changes of neurons in the spinal cord were investigated by the immunostaining of NeuN. Cell bodies and dendrites of large motor neurons in the ventral horn were clearly stained in control mice. The density of immunostaining in motor neurons was reduced in both the cervical and lumber spinal cord of A53T mice compared to control mice (Fig. 7e, f). EA prevented the loss of these neurons in the spinal cord.

### EA modulated expression of Nrf2, HO-1, and GCLM proteins in A53T mice

**Fig. 8:**
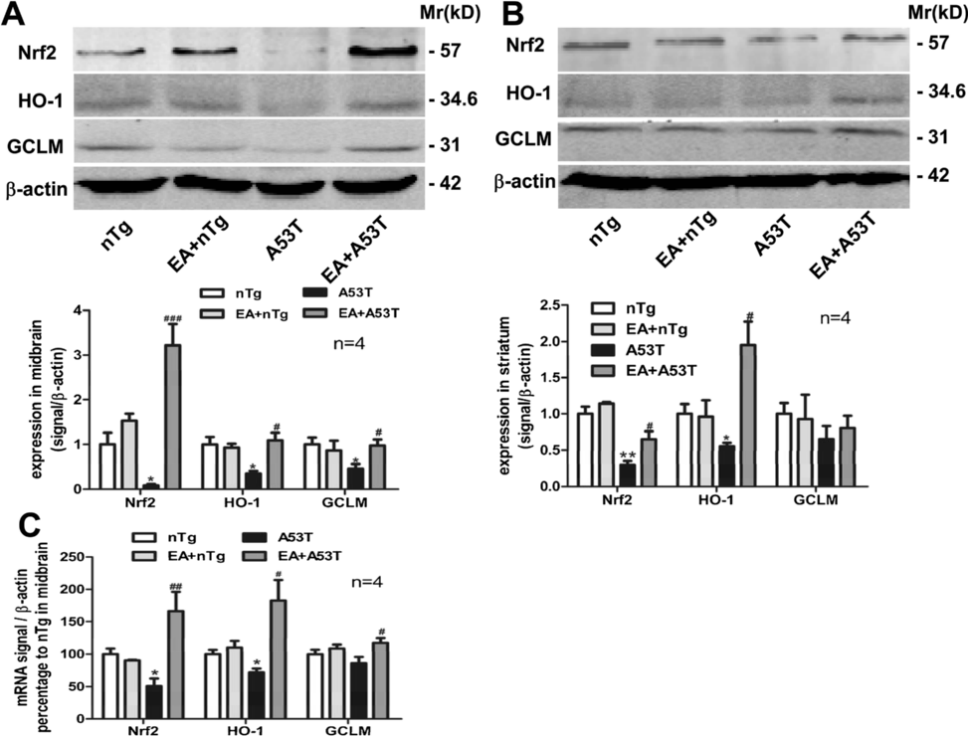
EA increased expression of Nrf2 and its downstream factors in the midbrain and striatum of A53T mice. **a** and **b** Effects of EA on Nrf2, HO-1 and GCLM protein expression in the midbrain (**a**) and striatum (**b**). Representative immunoblots are shown above the quantified data. Note that EA significantly reversed the reduction of three proteins in A53T mice. **c** Effects of EA on Nrf2, HO-1, and GCLM mRNA expression in the midbrain. EA produced the similar recovery of reduced Nrf2 and HO-1 mRNA expression in A53T mice. Data are expressed by means ± SEM (*n* = 4 per group). *P < 0.05 vs. nTg group. ##*P* < 0.01, ###*P* < 0.001vs. A53T group

It is well known that Nrf2 suppresses neurodegeneration via a mechanism involving the inhibition of oxidative stress and gliosis. In A53T mice, Nrf2 protein levels were dramatically reduced in the midbrain and striatum compared to nTg mice (Fig. 8a, b). EA stimulation substantially increased Nrf2 proteins in the midbrain to a level higher than that in nTg control mice (Fig. 8a). EA also increased Nrf2 expression in the striatum (Fig. 8b). Meanwhile, EA markedly elevated the Nrf2 mRNA level in the midbrain of A53T mice compared to untreated-mice (Fig. 8c). Similarly, EA treatment significantly increased HO-1 protein expression in the midbrain and striatum and GCLM expression in the midbrain (Fig. 8a, b). At the mRNA level, EA upregulated HO-1 and GCLM mRNA expression in the midbrain (Fig. 8c). These results indicate the ability of EA to upregulate expression of these proteins in both the midbrain and striatum of A53T mice.

## Discussion

The principal finding of the present study was that high frequency EA stimulation alleviated motor impairments and delayed the progression of disease in A53T α-syn transgenic mice. EA seems to be anti-inflammatory since it inhibited microglial activation, reduced cytokine production, and preserved the moderate astrogliosis in the brain. EA also increased expression of Nrf2-dependent antioxidants and prevented the loss of midbrain dopaminergic neurons and motor neurons in the spinal cord. These effects establish EA as a useful tool for treating neuroinflammatory disorders.

### EA modulated α-syn pathology and alleviated motor disability in A53T mice

According to Braak et al. [33], α-syn deposition occurs at the earliest stage of PD without neuronal loss or clinical symptoms. The deposition takes place in both astrocytes and microglia [10], which eventually leads to the characteristic behavioral and pathological features of PD [34]. In transgenic mice, aggregated and truncated forms of α-syn were significantly increased in the brain [15], which could impair the normal function of astrocytes and initiate the downstream pathogenic events [35]. In this study, we observed an accumulation of α-syn proteins in the midbrain and striatum of symptomatic A53T mice. Interestingly, α-syn expression was subject to the EA modulation. After prolonged EA stimulation, the amount of α-syn proteins in the midbrain and striatum was reduced. While α-syn protein levels were reduced, α-syn mRNAs were not affected by EA. This seems to indicate that EA may primarily affect posttranscriptional steps, such as translation, aggregation, degradation, and/or propagation, to alter α-syn protein expression. How EA regulates α-syn expression is unclear. It has been found that EA increased ubiquitin C terminal hydrolase-L1 (UCH-L1) and ubiquitin activating enzyme-1 (UBE1) expression in the SN, which led to a reduction of α-syn expression and toxicity [36]. Thus, EA likely modulates α-syn expression via a UCH-L1/UBE1-dependent mechanism. In addition, apolipoprotein D, a lipocalin involved in lipid metabolism, is constitutively expressed in astrocytes [37]. Due to structural similarities, lipocalins act as autocrine factors to interact with α-syn and reduce reactive astrogliosis [37]. Thus, it is possible that EA could modulate the substances such as lipocalins to indirectly influence α-syn and astrocytes.

The expression level of exogenous A53T α-syn determines the onset and severity of behavioral abnormalities in these mutant mice. It has been found that the early-onset paralysis occurred in A53T-E2 mice (the high expression of α-syn in astrocytes), and however, there were no obvious behavior abnormalities in A53T-A8 mice (the low expression line) [15]. Thus, the improving effect of EA on motor behaviors may be derived from the regulation of α-syn expression in A53T mice.

In A53T mice, excessive A53T α-syn in astrocytes disrupted the normal function of astrocytes, leading to inflammation and microglial activation in the midbrain and spinal cord. Activated microglia then trigger the loss of neuron, which may contribute to the movement disability. A recent study has found that conditional expression of tet-regulated A53T missense mutation α-syn in the midbrain dopaminergic neurons caused key motor and pathological phenotypes of PD [38], although not paralysis. This indicates that the paralysis phenotype results from the loss of motor neurons in the spinal cord. In this study, EA was effective in alleviating both PD-like motor behaviors and paralysis in A53T mice, which corresponds well with the EA effect in preventing the loss of dopaminergic neurons in the midbrain and motor neurons in the spinal cord. Taken together, EA reverses the loss of neurons in the midbrain and spinal cord to improve respective motor activities.

### Roles of reactive astrogliosis in PD and EA effects

Severe reactive astrogliosis interferes with the normal function of astrocytes that is critical for maintaining the integrity of blood–brain barrier (BBB) and for regulating the homeostasis of glutamate transporters [15]. While severe astrogliosis is disadvantageous, reactive astrocytes also play a compensatory role. This was evidenced by the finding that selective and conditional ablation of reactive astrocytes caused greater neuronal and oligodendrocyte degeneration, greater inflammatory responses, less recovery of the BBB, and more severe functional deficits in a spinal cord injury transgenic mouse [39]. Moreover, reactive astrocytes promoted dopaminergic neuronal recovery [40]. In our studies, A53T mice showed severe astrogliosis as evidenced by a substantial increase in the number of GFAP-positive astrocytes and GFAP protein overexpression in the SN. EA was able to partially reduce astrogliosis. However, it is important to note that EA did not completely abolish reactive astrogliosis. As a matter of fact, a moderate and significant degree of reactive astrogliosis remained following EA stimulation. Thus, EA seems to tune down the excessive reactive astrogliosis and at the same time preserve moderate reactive astrogliosis. This fine adjustment is useful to preserve a certain amount of reactive astrogliosis beneficial for neuronal survival.

The functional role of reactive astrocytes in regulating neuroinflammation is noteworthy [41, 10]. In A53T mice, reactive astrocytes are major cells that recruit and trigger the activation of microglia and increase expression of pro-inflammatory cytokines such as TNF-α and IL-1β in the midbrain and striatum. By activating microglia and releasing cytokines, astrocytes are linked to the loss of dopamine neurons and motor symptoms of PD. In support of this, we found that the number of activated microglia was enhanced in the SN of A53T mice. Parallel increases in pro-inflammatory cytokines (TNF-α and IL-1β) were also observed in the midbrain and striatum. Meanwhile, EA inhibited microglial activation and inflammatory cytokine expression. These results suggest that EA may exert its alleviating effects on PD symptoms through a mechanism involving the inhibition of microglia activation and microglia-mediated inflammatory reactions.

Of note, microglia activation is classified into two major phenotypes: detrimental M1 (classical activation) and beneficial M2 (alternative activation) phenotypes [42]. Astrocytic expression of A53T certainly evoked M1 microglial activation at the symptomatic stage. EA effectively reduced the activation of microglia and expression of M1-related inflammatory cytokines, such as TNF-α and IL-1β. Little is known about the activation of the M2 phenotype in the PD pathogenesis [43]. Recently, an epigenetic study has suggested that histone H3K27me3 demethylase Jmjd3 may facilitate the switch of microglia phenotypes from M2 to M1 polarization [44]. Therefore, it will be interesting to know whether EA helps switch M1/M2 phenotypes in the future studies.

### Nrf2 signal pathways in PD and EA effects

An important anti-oxidant and anti-inflammatory mechanism involves the Nrf2-ARE pathway. Selective Nrf2 expression in astrocytes is sufficient to prevent the 1-methyl-4-phenyl-1, 2, 3, 6-tetrahydropyridine-induced loss of dopaminergic neurons [40]. In response to exogenously added α-syn, Nrf2^−/−^ mice showed an exacerbated loss of nigral dopaminergic neurons and increased neuroinflammation and gliosis at the early PD stage [28]. Therefore, the activation of the Nrf2-ARE pathway is of a neuroprotective nature [45]. Consistent with this, Nrf2 modulated microglial activation to produce anti-inflammatory responses [46]. In contrast, impaired Nrf2 responses correlated with a shift in the microglial activation profile, towards an increased production of proinflammatory markers (IL-6, IL-1β, iNOS, and NF-κB). The relevance of Nrf2-regulated genes in inflammation has also been identified in postmortem biopsies of PD patients. An increase in HO-1 expression in glial cells has been revealed, which however was insufficient to elicit an effective protection of SN neurons [45]. In this study, we observed lower expression of Nrf2 and its downstream molecules (HO-1 and GCLM) in the midbrain and striatum of A53T mice. EA was able to upregulate expression of these proteins. Of note, the Nrf2 protection is not restricted to α-syn toxicity. In fact, Nrf2 plays a role in overall neuronal degeneration seen in oxidative stress, environmental toxins, proteinopathy, and inflammation [45]. Thus, Nrf2 represents a common site for developing pharmacotherapies to treat a wide range of neurodegenerative diseases, including PD, Alzheimer’s disease, and Huntington’s disease.

### Potential anti-inflammation pathways of EA

Neural mechanisms underlying the EA effect have long been investigated [24, 47, 25]. Recent studies suggest a neural-immune model. EA is typically applied to the deep tissue enriched with sensory innervations [48]. Sensory signals induced by EA stimulation alter the immune system to dynamically control immunity activity [49]. Indeed, EA stimulation at the ST36 Zusanli acupoint decreased the lipopolysaccharide-induced inflammatory cytokines, including TNF, monocyte chemotactic protein-1, IL-6, and interferon-γ [24]. However, the anti-inflammatory action of EA was abolished by surgical sectioning of the sciatic nerve. Conversely, direct stimulation of the sciatic nerve mimicked the anti-inflammatory effect of EA [24]. Thus, it appears that EA stimulation of the acupoints on the hind-limb acts as an effective vagal stimulation, which can control inflammation. However, the exact anatomical pathways connecting EA signals to the immune system are poorly understood [50]. Future studies will explore those pathways in details.

## Conclusions

Our results demonstrate that EA effectively increased survival of A53T mice, decreased non-neuronal expression of α-syn, and inhibited inflammatory responses. Meanwhile, EA preserved the moderate astrogliosis, protected the dopaminergic neurons, and alleviated motor impairments. These data indicate a causative relationship between glial α-syn-mediated inflammation responses and chronic dopaminergic neurodegeneration. Moreover, EA by inhibiting degenerative processes at multiple levels has potential to be a useful tool for treating neurodegenerative diseases.

## Abbreviations

α-syn: α-synuclein
ANOVA: analysis of variance
ARE: Antioxidant response element
BBB: Blood–brain barrier
DAPI: 4′,6-diamidino-2-phenylindole
DOX: Doxycycline
EA: Electroacupuncture
ELISA: Enzyme-linked immunosorbent assay
GCLM: Glutamate-cysteine ligase modifier subunits
GFAP: Glial fibrillary acidic protein
HO-1: Heme oxygenase-1
Iba1: Ionized calcium-binding adaptor molecule-1
IL-1β: Interleukin-1β
IL-6: Interleukin-6
NeuN: Neuronal nuclei
Nrf2: Nuclear factor E2-related factor 2
PCR: Polymerase chain reaction
PD: Parkinson’s disease
PBS: Phosphate buffered saline
SN: Substantia nigra
SNpc: Substantia nigra pars compacta
tetO: Tetracycline operator
TH: Tyrosine hydroxylase
TNF-α: Tumor necrosis factor-α
UBE1: Ubiquitin activating enzyme-1
UCH-L1: Ubiquitin C terminal hydrolase-L1
